# Supplementary figures and images for: The CATP-8/P5A-type ATPase functions in multiple pathways during neuronal patterning
Source: PLoS Genet. 2021 Jul 1;17(7):e1009475. doi: 10.1371/journal.pgen.1009475 (PMC8279360; doi:10.1371/journal.pgen.1009475)

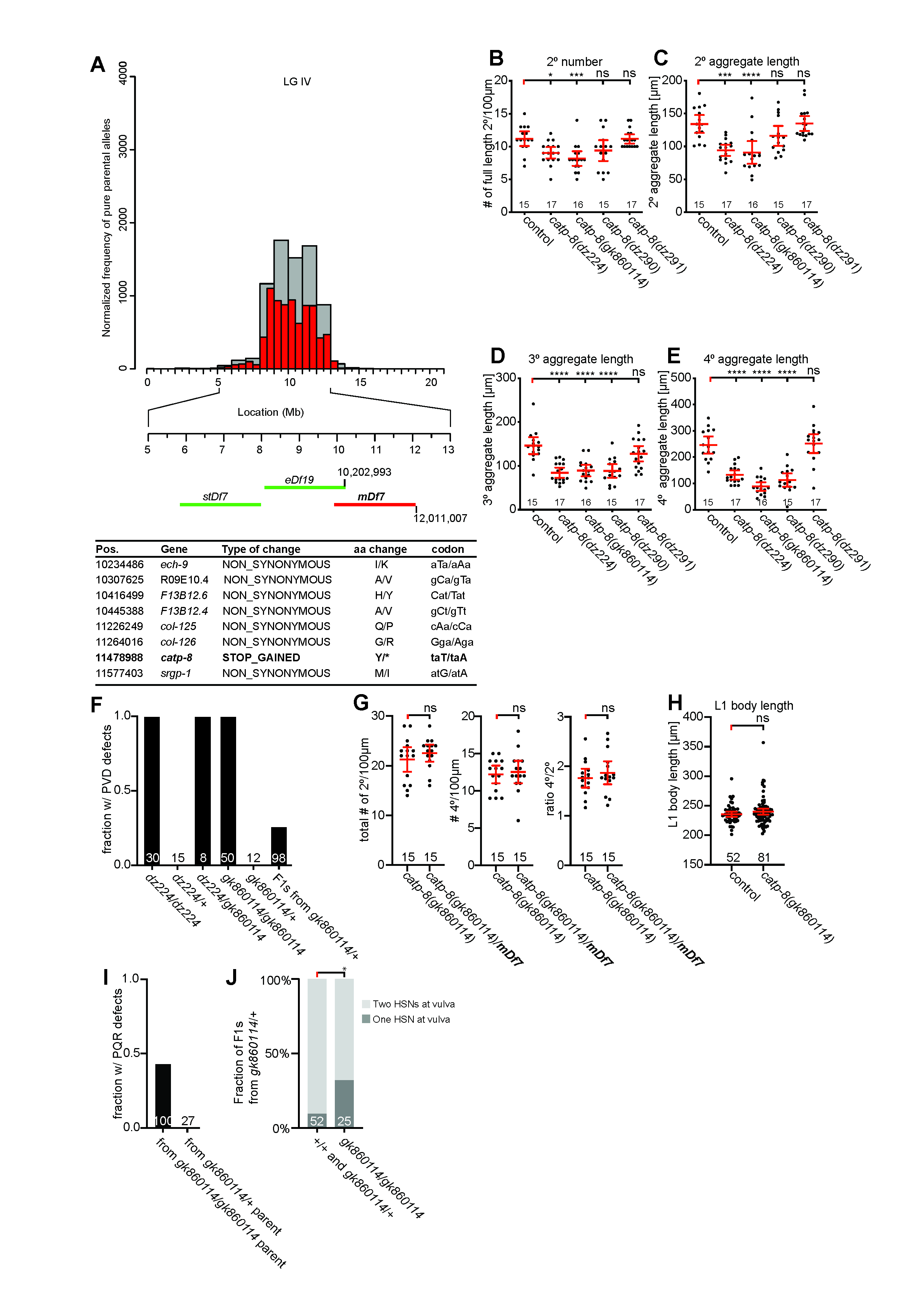

Supplement: S1 Fig — A. Frequency plot of N2 SNPs from whole genome sequencing results as per Hawaiian SNP mapping [26], indicating dz224 lesions reside between 6 Mbp and 13 Mbp on linkage group (LG) IV. Deficiencies covering the region are indicated below (green, complementation of dz224; red non-complementation of dz224), demonstrating that dz224 lesions lies between 10,202,993, and 12,011,007 bp, the physical positions of the last known genes inside of eDf19 and the first one outside of mDf7, respectively. The table below lists lesions detected within this range, with catp-8 containing the only stop-gain mutation. B.—E. Quantification of the number of secondary branches that reach the tertiary line where muscle and epidermis abut (B)(i.e. excluding ectopic stunted secondary branches), aggregate length of all secondary (C), tertiary (D), and quaternary (E) dendrite branches 100 μm anterior to the PVD cell body in the genotypes indicated. Data are represented as the mean ± 95% confidence interval. Statistical significance was calculated using one-sided ANOVA with Tukey’s multiple comparison test. * P ≤ 0.05, *** P ≤ 0.001, **** P ≤ 0.0001, ns not significant. F. Complementation assay tallying the percentage of animals with PVD defects. Note that there is non-complementation between dz224 and catp-8(gk860114), recessiveness of dz224 and catp-8(gk860114), and absence of maternal effects. G. Quantification of the number of quaternary and secondary branches, and the ratio of quaternary to secondary branches 100 μm anterior to the PVD cell body in catp-8(gk860114) and catp-8(gk860114)/mDf7 transheterozygous animals. No enhancement was observed in the transheterozygous genotype, showing that gk860114 behaves as a genetic null allele. Data are represented as the mean ± 95% confidence interval. ns not significant, Kruskal-Wallis test with Dunn’s multiple comparisons test. n = 14 animals per genotype. H. Body length of wildtype and catp-8(gk860114) L1 larval animals, measured and presented as m [file pgen.1009475.s007.tiff]

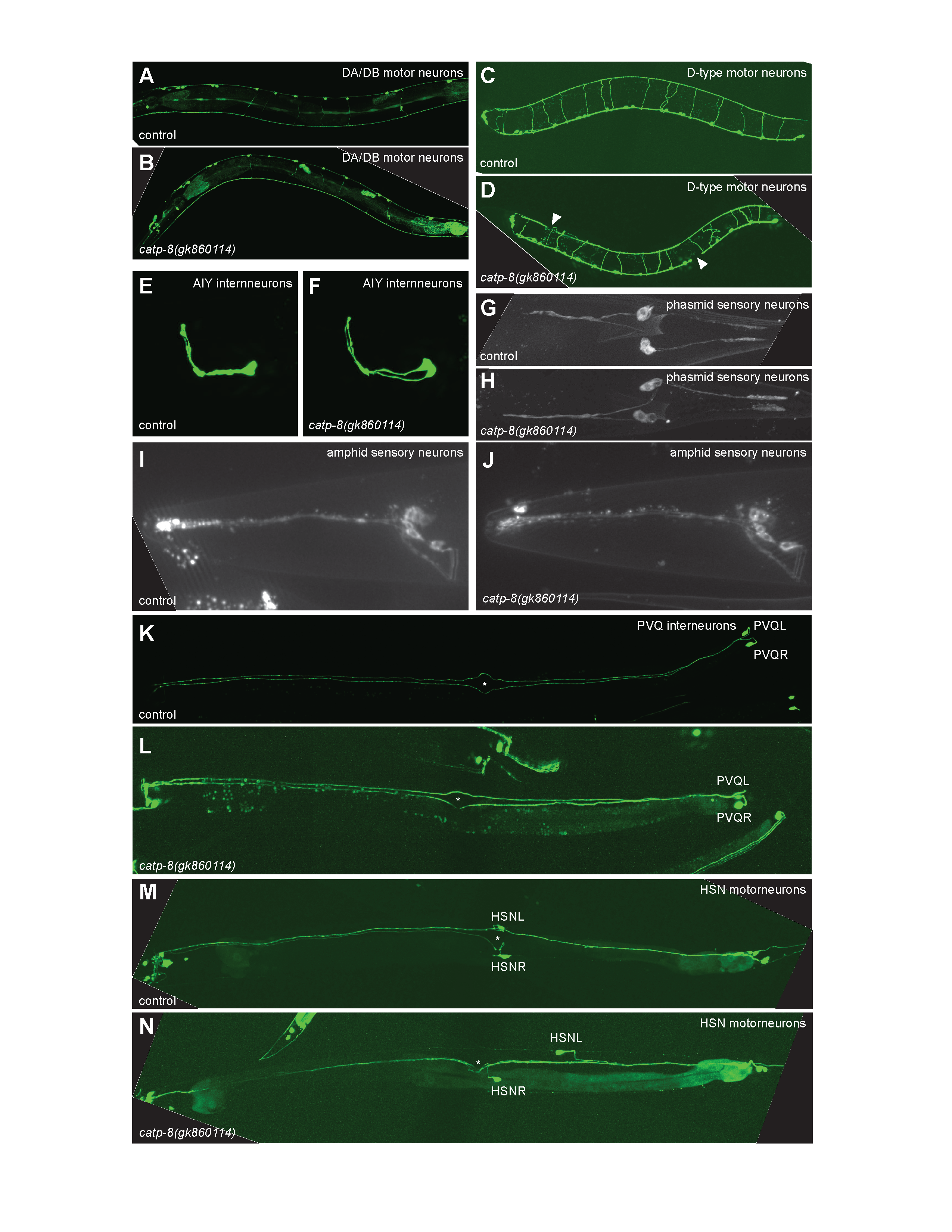

Supplement: S2 Fig — A.—B. Maximum z-projection confocal images of DA/DB cholinergic motor-neurons (visualized by evIs82b (Is[unc-129p::GFP]) of wildtype (A) and catp-8 mutant animals (B). No difference between the genotypes were observed. Lateral views, ventral up, anterior to the left. C.—D. Maximum z-projection confocal images of D-type GABAergic motor-neurons (visualized by juIs76(Is[unc-25p::GFP]) of wild type (C) and catp-8 mutant animals (D). Occasional gaps caused by under-extension of neurites can be observed in catp-8 mutant animals. Lateral views, dorsal up, anterior to the left. E.—F. Maximum z-projection confocal images of AIY interneurons (visualized by mgIs32 (Is[ttx-3p::GFP]) in wildtype (E) and catp-8 mutant animals (F). No difference between the genotypes was observed. Lateral views, dorsal up, anterior to the left. G.—J. Maximum z-projection apotome images of DiI stained phasmid and amphid sensory neurons, respectively in wildtype (G,I) and catp-8 mutant animals (H,K). No difference between the genotypes was observed. Ventral views, anterior to the left. Lateral views, dorsal up, anterior to the left. K.—N. Maximum z-projection confocal images of PVQ interneurons (visualized by oyIs14 (Is[sra-6p::GFP]) (K,L) and HSN motor-neurons (visualized by dzIs75 (Is[kal-9p::GFP]) (M,N) in wildtype and catp-8 mutant animals. No defects were observed in PVQ axons at the midline, but HSN neurons showed a significant number of midline cross overs in catp-8 mutant animals (see also Table 1). Ventral views, anterior to the left. (TIFF) [file pgen.1009475.s008.tiff]

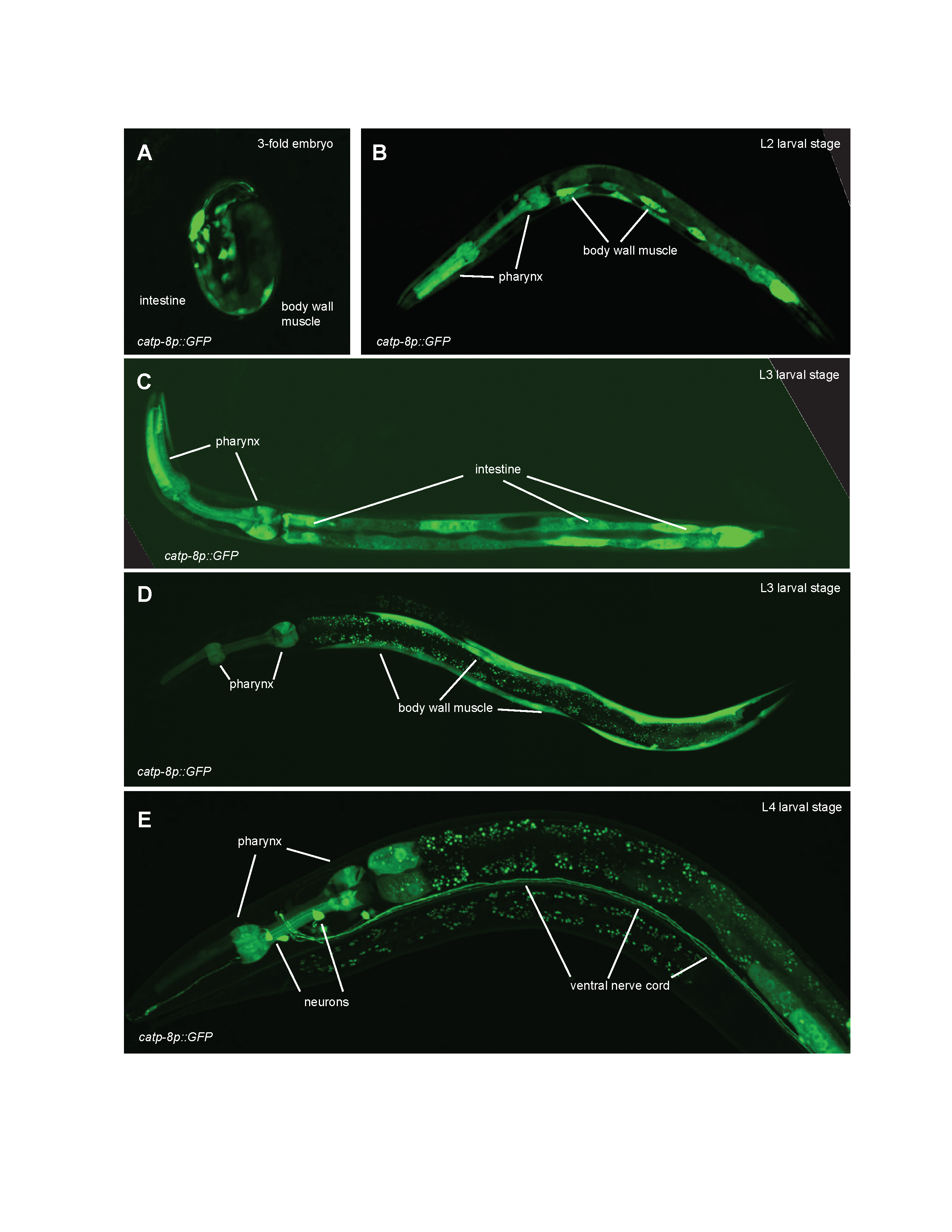

Supplement: S3 Fig — A.—E. Maximum z-projection confocal images of an embryo (A), L2 larval animal (B), L3 larval animal (C-D) and L4 larval animal (E) carrying the catp-8p promoter GFP reporter fusion array dzEx2101. dzEx2101 exhibits a high degree of mosaicism, resulting in staining of different tissues across the population, including muscle, intestine, pharynx, and some unidentified neurons. (TIFF) [file pgen.1009475.s009.tiff]

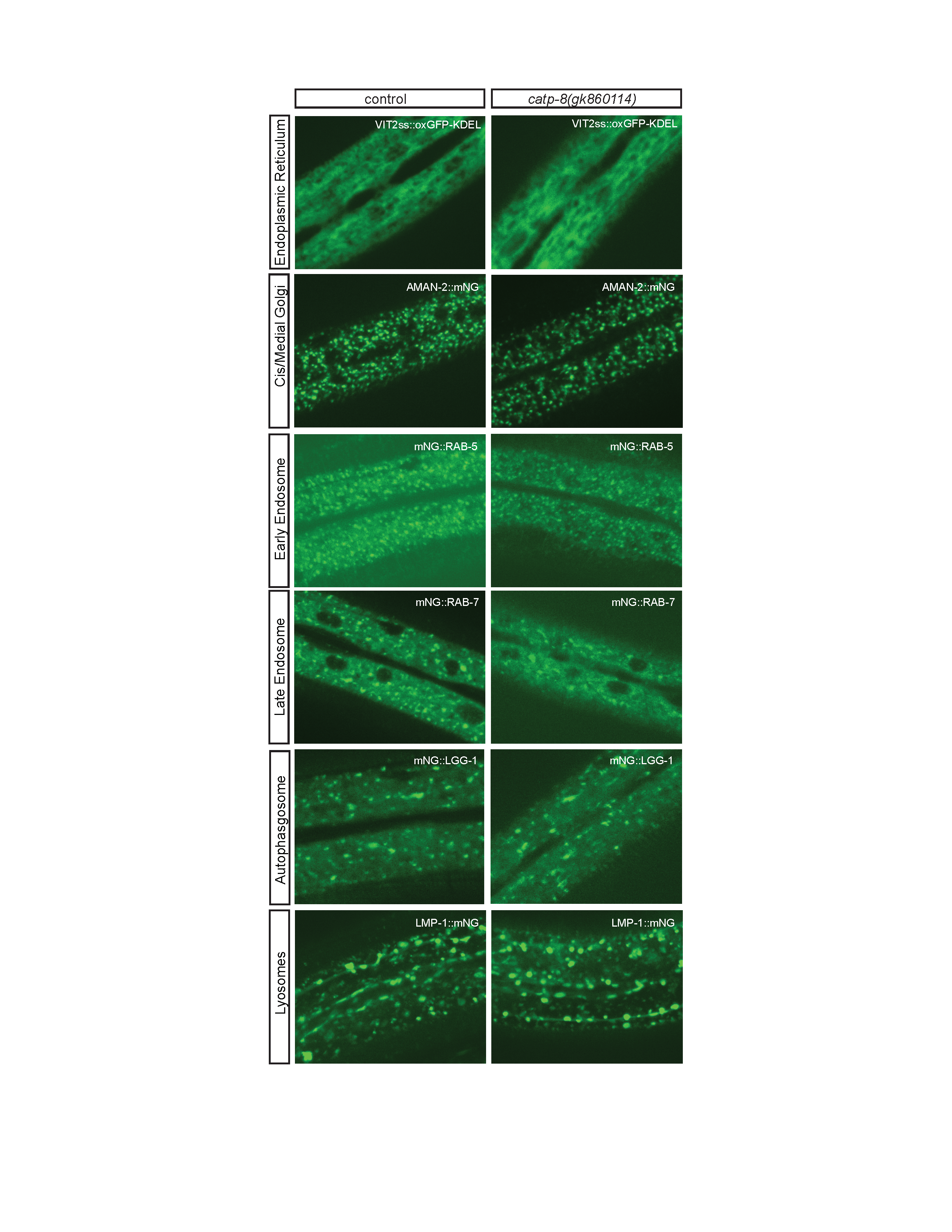

Supplement: S4 Fig — Confocal images of single copy insertion transgenes of epidermally expressed organellar reporters in catp-8(gk860114) mutant and wild type animals, for ER/endoplasmic reticulum (pwSi82 [Phyp-7::VIT2ss::oxGFP-KDEL]), cis/medial Golgi (pwSi202 [Phyp7::AMAN-2::mNG;HYG-R]), early endosomes (pwSi145 [Phyp7::mNG::RAB-5;HYG-R]), late endosomes and lysosomes pwSi140 [Phyp7::mNG::RAB-7;HYG-R]), autophagosomes (pwSi144 [Phyp7::mNG::LGG-1;HYG-R]), and lysosomes (pwSi205 [Phyp7::LMP-1::mNG;HYG-R]). (TIFF) [file pgen.1009475.s010.tiff]

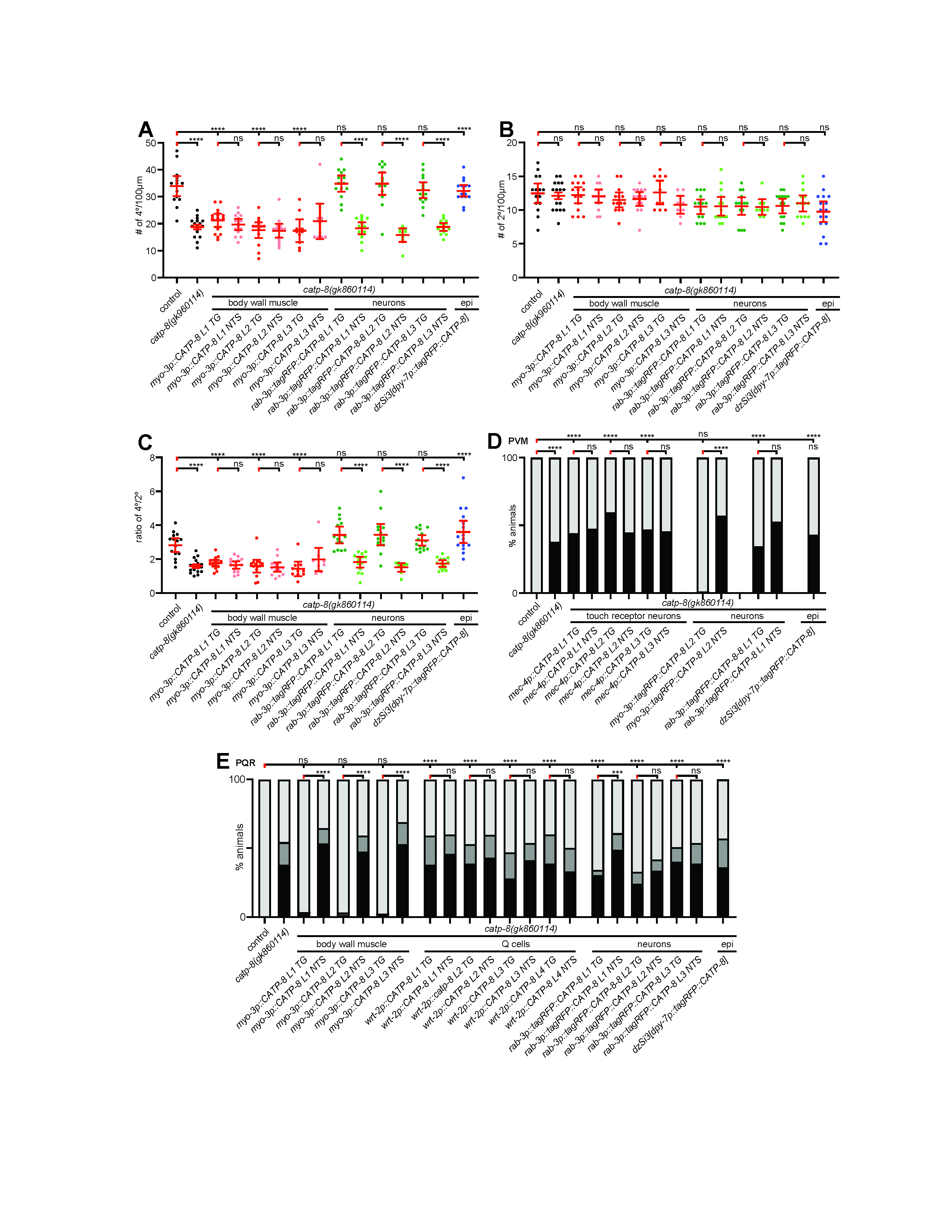

Supplement: S5 Fig — A.—C. Quantification of the number of quaternary branches (A), secondary branches (B), and the ratio of quaternary to secondary branches (C) 100 μm anterior to the PVD cell body in animals of all extrachromosomal rescue lines tested. TG: transgenic animals, NTS: non-transgenic siblings. Data are represented as the mean ± 95% confidence interval. **** P < 0.0001, ns not significant, Kruskal-Wallis test with Dunn’s multiple comparisons test. n = 14 animals per genotype. D.—E. Percentage of animals with the indicated migration phenotype of PVM (D) and PQR (E) for all extrachromosomal rescue lines tested. TG: transgenic animals, NTS: non-transgenic siblings. **** P < 0.0001, ns not significant, Chi-squared test. n > 75 animals per genotype. (TIFF) [file pgen.1009475.s011.tiff]

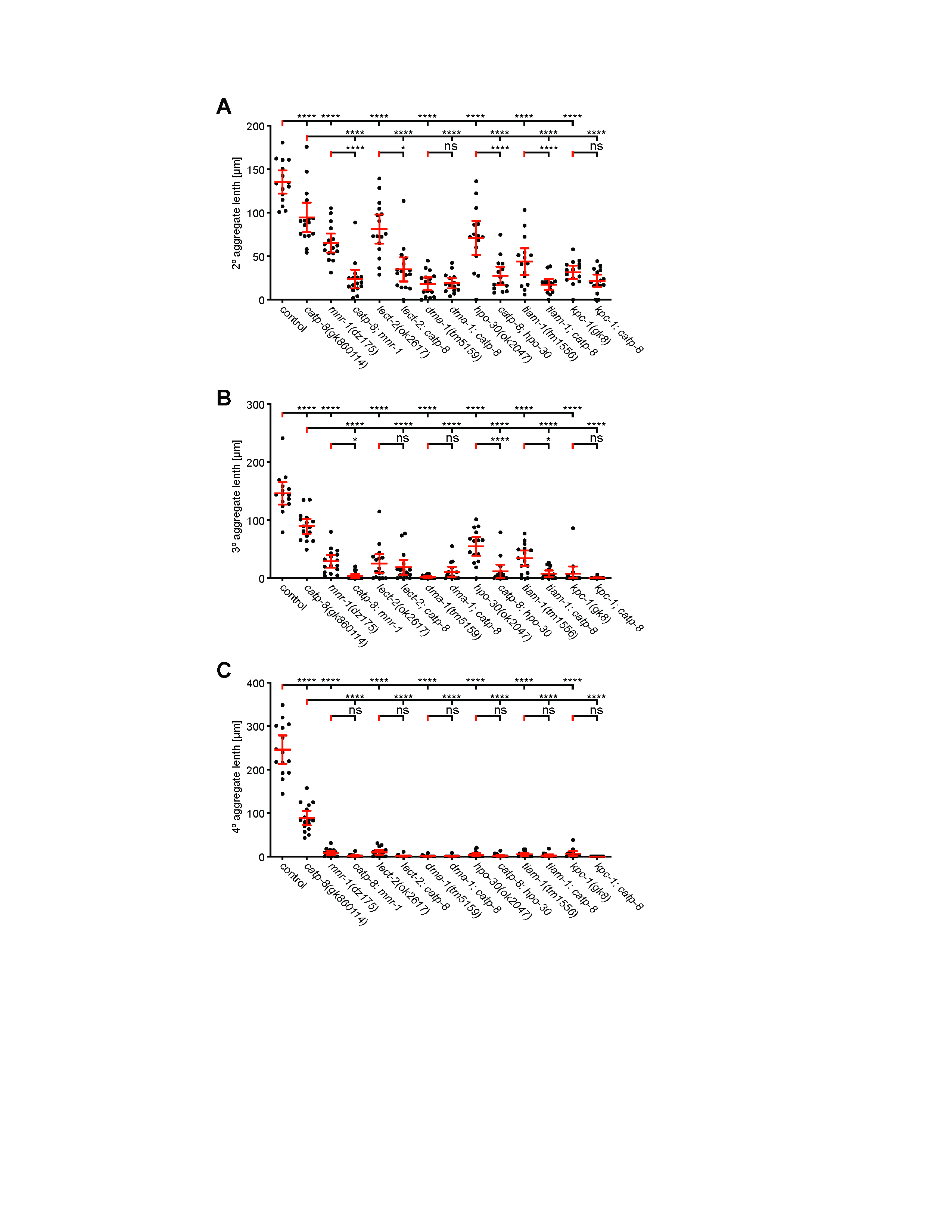

Supplement: S6 Fig — A.—C. Quantification of the aggregate length of secondary (A), tertiary (B), and quaternary (C) dendrite branches within 100μm anterior to the PVD cell body in animals of the indicated genotypes. Note that all alleles are molecular or genetic null alleles. Data are represented as the mean ± 95% confidence interval. * P < 0.05, ** P < 0.01, *** P < 0.001, **** P < 0.0001 ns not significant; one-sided ANOVA with Tukey’s multiple comparison test. n = 15 animals per genotype. (TIFF) [file pgen.1009475.s012.tiff]

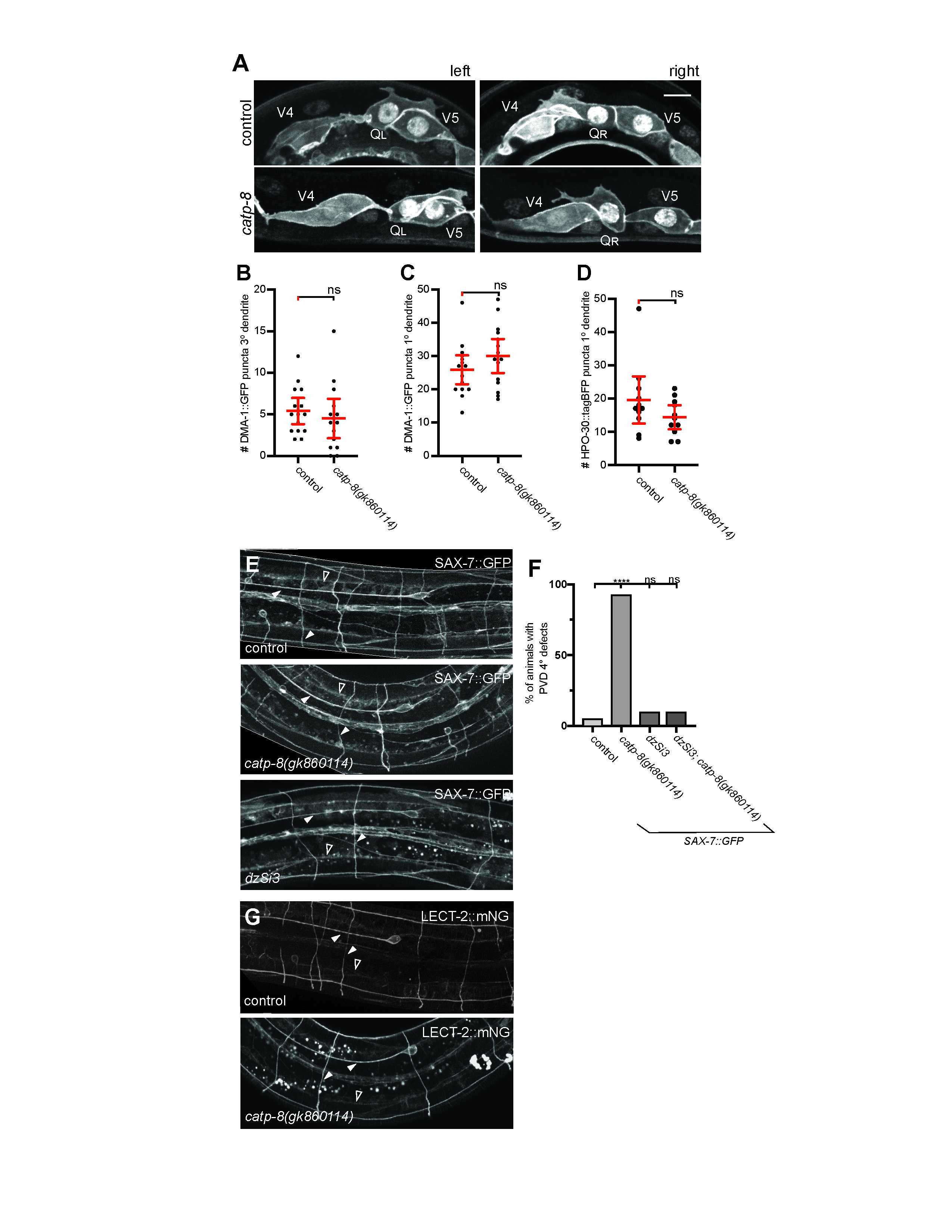

Supplement: S7 Fig — A. Fluorescent images of initial Q neuroblasts morphologies, when polarization is just beginning in control and catp-8(gk860114) mutant animals, imaged 1.5 hours after hatching. Left and right sides of the animals are shown as indicated. Scale bar = 5 μm. B.—C. Quantification of DMA-1::GFP puncta either in the primary (B) or on the tertiary (C) dendrite 100μm anterior of the cell body in the genotypes indicated. ns, not significant, Mann-Whitney test. n = 14 animals per genotype. D. Number of HPO-30::tagBFP puncta in the primary dendrite 100 μm anterior to the cell body in control or catp-8 mutant animals, presented as mean ± 95% confidence interval. ns not significant, Mann-Whitney test. n = 10 animals per genotype. E. Fluorescent images of animals expressing a functional fosmid based SAX-7::GFP reporter (ddIs290) in control, catp-8(gk860114) mutants, and animals overexpressing catp-8 in the epidermis (dzSi3). White arrowheads denote neuronal staining, while open arrows indicate examples of epidermal staining at the lateral epidermal ridge. F. Quantification of the percentage of animals with PVD 4° branching defects in the indicated genotypes in a ddIs290 background. Statistical comparisons were performed using the Z-test. ****, P < 0.0005, ns not significant. n = 15 animals per genotype. G. Fluorescent images of animals expressing lect-2::mNG endogenous knock-in (dz249) [38] in control and catp-8(gk860114) mutant animals. White arrowheads denote neuronal staining, while open arrows indicate examples of epidermal staining at the lateral epidermal ridge. (TIFF) [file pgen.1009475.s013.tiff]
